# Genotype-specific spinal cord damage in spinocerebellar ataxias: an ENIGMA-Ataxia study

**DOI:** 10.1136/jnnp-2023-332696

**Published:** 2024-02-21

**Authors:** Thiago Junqueira Ribeiro Rezende, Isaac Adanyaguh, Orlando G P Barsottini, Benjamin Bender, Fernando Cendes, Leo Coutinho, Andreas Deistung, Imis Dogan, Alexandra Durr, Juan Fernandez-Ruiz, Sophia L Göricke, Marina Grisoli, Carlos R Hernandez-Castillo, Christophe Lenglet, Caterina Mariotti, Alberto R M Martinez, Breno K Massuyama, Fanny Mochel, Lorenzo Nanetti, Anna Nigri, Sergio E Ono, Gülin Öz, José Luiz Pedroso, Kathrin Reetz, Matthis Synofzik, Helio Teive, Sophia I Thomopoulos, Paul M Thompson, Dagmar Timmann, Bart P C van de Warrenburg, Judith van Gaalen, Marcondes C França, Ian H Harding

**Affiliations:** 1 Department of Neurology, University of Campinas (UNICAMP), Campinas, Brazil; 2 Brazilian Institute of Neuroscience and Neurotechnology, Campinas, Brazil; 3 Center for Magnetic Resonance Research, Department of Radiology, University of Minnesota, Minneapolis, Minnesota, USA; 4 Department of Neurology, Federal University of São Paulo, São Paulo, SP, Brazil; 5 Department of Diagnostic and Interventional Neuroradiology, University Hospital Tübingen, Tübingen, Germany; 6 Graduate program of Internal Medicine, Internal Medicine Department, Hospital de Clínicas, Federal University of Paraná, Curitiba, Brazil; 7 University Clinic and Outpatient Clinic for Radiology, Department for Radiation Medicine, University Hospital Halle (Saale), University Medicine Halle, Halle (Saale), Germany; 8 Department of Neurology, RWTH Aachen University, Aachen, Germany; 9 JARA-BRAIN Institute Molecular Neuroscience and Neuroimaging, Research Center Jülich GmbH, Jülich, Germany; 10 Sorbonne Université, Paris Brain Institute (ICM), Pitié-Salpêtrière Hospital, AP-HP, INSERM, CNRS, University Hospital Pitié-Salpêtrière, Paris, France; 11 Neuropsychology Laboratory, Department of Physiology, Faculty of Medicine, National Autonomous University of Mexico, Mexico City, Mexico; 12 Institute of Diagnostic and Interventional Radiology and Neuroradiology and Center for Translational Neuro- and Behavioral Sciences (C-TNBS), Essen University Hospital, University of Duisburg-Essen, Essen, Germany; 13 Department of Neuroradiology, Fondazione IRCCS Istituto Neurologico Carlo Besta, Milan, Italy; 14 Faculty of Computer Science, Dalhousie University, Halifax, Nova Scotia, Canada; 15 Unit of Medical Genetics and Neurogenetics, Fondazione IRCCS Istituto Neurologico Carlo Besta, Milan, Italy; 16 Assistance Publique-Hôpitaux de Paris, Pitié-Salpêtrière University Hospital, Paris, France; 17 Clínica DAPI – Diagnóstico Avançado Por Imagem, Curitiba, Brazil; 18 Department of Neurodegenerative Diseases, Hertie Institute for Clinical Brain Research, Tübingen, Germany; 19 German Center for Neurodegenerative Diseases (DZNE), Tübingen, Germany; 20 Movement Disorders Unit, Neurology Service, Internal Medicine Department, Hospital de Clínicas, Federal University of Paraná, Curitiba, Brazil; 21 Imaging Genetics Center, Mark and Mary Stevens Institute for Neuroimaging and Informatics, Keck School of Medicine, University of Southern California, Marina del Rey, CA, USA; 22 Department of Neurology and Center for Translational Neuro- and Behavioral Sciences (C-TNBS), Essen University Hospital, University of Duisburg-Essen, Essen, Germany; 23 Department of Neurology, Donders Institute for Brain, Cognition, and Behaviour, Radboud University Medical Center, Nijmegen, Netherlands; 24 Department of Neurology, Rijnstate Hospital, Arnhem, Netherlands; 25 QIMR Berghofer Medical Research Institute, Brisbane, Queensland, Australia; 26 Department of Neuroscience, Central Clinical School, Monash University, Melbourne, Victoria, Australia; 27 Monash Biomedical Imaging, Monash University, Clayton, Victoria, Australia

**Keywords:** MRI, cerebellar ataxia, image analysis, neurogenetics

## Abstract

**Background:**

Spinal cord damage is a feature of many spinocerebellar ataxias (SCAs), but well-powered in vivo studies are lacking and links with disease severity and progression remain unclear. Here we characterise cervical spinal cord morphometric abnormalities in SCA1, SCA2, SCA3 and SCA6 using a large multisite MRI dataset.

**Methods:**

Upper spinal cord (vertebrae C1–C4) cross-sectional area (CSA) and eccentricity (flattening) were assessed using MRI data from nine sites within the ENIGMA-Ataxia consortium, including 364 people with ataxic SCA, 56 individuals with preataxic SCA and 394 nonataxic controls. Correlations and subgroup analyses within the SCA cohorts were undertaken based on disease duration and ataxia severity.

**Results:**

Individuals in the ataxic stage of SCA1, SCA2 and SCA3, relative to non-ataxic controls, had significantly reduced CSA and increased eccentricity at all examined levels. CSA showed large effect sizes (*d*>2.0) and correlated with ataxia severity (r<−0.43) and disease duration (r<−0.21). Eccentricity correlated only with ataxia severity in SCA2 (r=0.28). No significant spinal cord differences were evident in SCA6. In preataxic individuals, CSA was significantly reduced in SCA2 (*d*=1.6) and SCA3 (*d*=1.7), and the SCA2 group also showed increased eccentricity (*d*=1.1) relative to nonataxic controls. Subgroup analyses confirmed that CSA and eccentricity are abnormal in early disease stages in SCA1, SCA2 and SCA3. CSA declined with disease progression in all, whereas eccentricity progressed only in SCA2.

**Conclusions:**

Spinal cord abnormalities are an early and progressive feature of SCA1, SCA2 and SCA3, but not SCA6, which can be captured using quantitative MRI.

**WHAT IS ALREADY KNOWN ON THIS TOPIC**

- Spinal cord degeneration is thought to be a key aspect of many spinocerebellar disorders, but in vivo studies are surprisingly lacking with existing MRI studies only available for small cohorts of individuals with SCA1 and SCA3. Here, we leverage the ‘big data’ potential of the ENIGMA-Ataxia consortium to undertake by-far the largest and most comprehensive assessment of cervical spinal cord morphometry in the most common spinocerebellar ataxias (SCAs; SCA1, SCA2, SCA3, and SCA6).

**WHAT THIS STUDY ADDS**

- Cross-sectional area (CSA) was already reduced in preataxic individuals with SCA2, and SCA3, with similar trends in SCA1. CSA also presented very large effect sizes, significant correlations with ataxia severity and symptom duration and progressive pattern of degeneration. Similarly, eccentricity was also increased in these groups, but showed significant correlation with ataxia severity and progressive degeneration only for the SCA2 cohort. Spinal cord morphometry does not appear to be impacted in SCA6.

**HOW THIS STUDY MIGHT AFFECT RESEARCH, PRACTICE OR POLICY**

- Quantitative spinal cord MRI contributes to the understanding of genotype–phenotype correlations in SCA1, SCA2, SCA3 and SCA6 and uncover the potential use of CSA as biomarkers for clinical use, but not to SCA6 which demonstrate the ‘pure’ cerebellar conceptions of this disease.

## Introduction

Spinocerebellar ataxias (SCAs) are a heterogeneous group of autosomal dominant neurodegenerative disorders that share gait and limb ataxia as the core clinical features. The most prevalent types of SCA (SCA1, SCA2, SCA3 and SCA6) are caused by CAG repeat expansions in the coding regions of the *ATXN1*, *ATXN2*, *ATXN3* and *CACNA1A* genes, respectively.^1 2^ Although progressive ataxia is the major clinical sign of all SCAs, there are also disease-specific clinical and neuropathological features.^3 4^ SCA1 shows the fastest progression and is pathologically characterised by pontine and cerebellar atrophy.^5^ In contrast, SCA2 shows more widespread and severe brain and cerebellar atrophy when compared with other SCAs.^6^ SCA3 is the most common SCA worldwide, and is primarily characterised by striatal and cerebellar atrophy in neuropathological studies.^7^ Lastly, SCA6 presents with a more restricted cerebellar atrophy, later ataxia onset and slower progression when compared with other SCAs.^8^

Neuroimaging studies in SCAs have largely focused on brain and cerebellar damage to-date.^9–17^ However, the spinal cord is increasingly recognised as an important structure in the pathogenesis of several SCAs.^4^ Indeed, Tezenas du Montcel and colleagues showed that pyramidal signs and posterior column signs precede ataxia in SCA1 and SCA3, suggesting that spinal cord is damaged early in these disorders.^18^ Spinal MRI research undertaken in SCAs to-date has been restricted to a small number of studies that have relied on modest sample sizes, limiting the sensitivity, reliability and generalisability of available evidence.^11–13 15 17 19^ Furthermore, magnitude of spinal cord damage in each SCA and how it progresses along the disease course, including in preataxic disease stages, remains unknown. These are important questions not only to elucidate the pathophysiology of SCAs, but also to uncover potential sensitive imaging biomarkers for future clinical trials.

**Figure 1:**
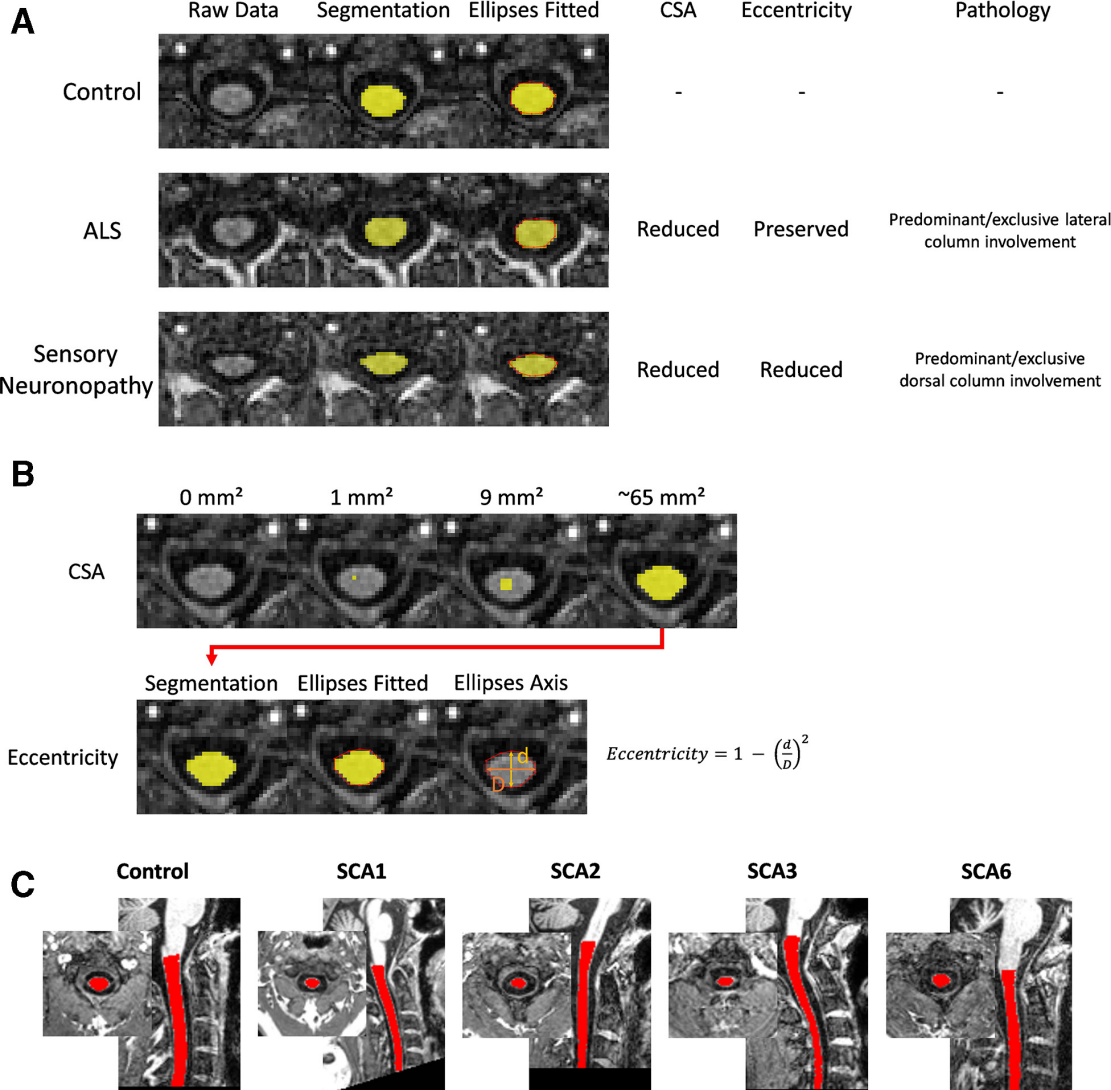
Schematic illustration of the spinal cord morphometric parameters used in this study. (A) Clinical correlates of these parameters. In diseases characterised by selective lateral column/corticospinal tract degeneration, there is CSA reduction, but preserved eccentricity (middle lane, patient with amyotrophic lateral sclerosis,ALS). In diseases characterised by selective dorsal column degeneration, there is combined CSA and eccentricity reduction (lower lane, patient with autoimmune sensory neuronopathy). All segmentations are shown in axial slices of the same spinal cord level (C2). (B) Computation of CSA and eccentricity. (C) An exemplar MRI with spinal cord mask from each cohort. CSA, cross-sectional area.

Quantification of spinal cord damage using in vivo MRI may provide new insights and novel opportunities for disease characterisation, treatment targeting and/or treatment monitoring in these diseases.^12 15^ Indeed, morphometric analyses of spinal cord MRI—looking at cross-sectional area (CSA) and eccentricity—have proven useful in other degenerative disorders.^20–22^ In particular, comparing diseases characterised by predominant/exclusive lateral column involvement, such amyotrophic lateral sclerosis or pure hereditary spastic paraplegia, versus diseases with predominant/exclusive dorsal column involvement, such as acquired sensory neuronopathies, provides an interpretive framework to interpret MRI spinal cord changes (figure 1A). Specifically, both groups of diseases present CSA reduction, but increased eccentricity is only reported in the latter. Hence, it is possible to hypothesise that eccentricity is a surrogate MRI marker for dorsal column involvement. In contrast, CSA reduction is unspecific and may be related to degeneration of both lateral and dorsal columns.

The ENIGMA-Ataxia working group is an international collaboration for aggregation and analysis of global multisite MRI datasets, including individuals with SCA. This platform allows for in-depth analyses that would not be feasible in single-centre studies. Using the ENIGMA-Ataxia platform, the main goals of this study were to characterise cervical spinal cord damage by assessing CSA and eccentricity in the most common SCAs (SCA1, SCA2, SCA3 and SCA6). Furthermore, we investigate the clinical correlates of spinal cord morphometric abnormalities in each SCA subtype, and provide insights into the progression of these features by comparing disease subgroups stratified by time from ataxia onset and disease severity.

## Methods

We performed a cross-sectional analysis of MRI data from nine sites within the ENIGMA-Ataxia working group. A total of 423 patients with molecular confirmation of SCA (75 SCA1, 102 SCA2, 192 SCA3 and 54 SCA6) and 398 age, sex and site-matched non-ataxic control subjects (70 for SCA1, 101 for SCA2, 178 for SCA3 and 49 for SCA6, respectively) were included (table 1, online supplemental tables S1–S4). Of these 423 patients with SCA, 59 had SARA score <3 at the time of MRI assessment (11 SCA1, 9 SCA2, 36 SCA3 and 3 SCA6; online supplemental table S5) and were classified as preataxic mutation carriers.^23–25^ For participants with ataxia, age at onset of gait ataxia symptoms, time since gait ataxia symptom onset and ataxia severity quantified using the Scale for Assessment and Rating of Ataxia (SARA) were recorded.^26^ For preataxic individuals, time to ataxia onset was estimated using the Tezenas formulas for SCA1 and SCA2^23^ and Peng formula for SCA3.^27^ For the SCA6 cohort, only three subjects were classified as preataxic, which was not sufficient for quantitative subgroup analysis. The diagnosis of SCA1, SCA2, SCA3 or SCA6 was genetically confirmed at all sites, but individual CAG repeat length was not available for all sites because of local reporting procedures or data privacy considerations.

10.1136/jnnp-2023-332696.supp1 Supplementary data

**Table 1:** Demographic, clinical and genetic data of the ataxic study participants*

		Average age, years (range)	Sex, n (male/female)	CAG repeat length, long allele	Average SARA (range)	Average time from ataxia onset, years (range)	Preataxic, n
SCA1	Controls (n=70)	46.1±12.4 (19–75)	39/31	–	–	–	–
	Patients (n=75)	45.0±12.2 (18–68)	40/35	45.4±4.8 (39–62)	11.8±6.9 (0–28)	7.7±5.9 (0–23)	11
SCA2	Controls (n=101)	42.7±14.1 (10–75)	51/49†	–	–	–	–
	Patients (n=102)	43.6±13.5 (9–70)	49/51†	40.8±4.8 (32–66)	13.9±8.8 (0–39)	9.7±6.7 (0–26)	9
SCA3	Controls (n=178)	46.7±12.5 (18–75)	92/86	–	–	–	–
	Patients (n=192)	47.2±12.6 (18–78)	96/96	70.3±4.3 (50–83)	11.3±8.2 (0–38)	9.2±2.3 (0–34)	36
SCA6	Controls (n=49)	60.5±11.9 (30–78)	31/18	–	–	–	–
	Patients (n=54)	63.9±9.9 (39–85)	30/23†	23.3±1.3 (21–27)	12.8±7.2 (0–26)	12.0±6.9 (0–30)	3

*Demographic data per group/site and for the preataxic participants are available in online supplemental tables S1–S5.

†Sex data unavailable for one or two individuals.

SARA, Scale for Assessment and Rating of Ataxia; SCA, spinocerebellar ataxia.

High-resolution 3D T1-weighted MRIs centred on the brain and including the upper cervical vertebrae were used to assess cervical spinal cord morphometry. All MRIs were acquired on 3 Tesla (T) clinical scanners with 1 mm isotropic spatial resolution (online supplemental table S6). All sites included a non-ataxic healthy control group with data acquired using the same protocol. Data collection, analysis and contributions to this project were governed by the human research ethics body at each site.

### Image processing

Data processing was undertaken using harmonised, previously published and public protocols developed by the ENIGMA-Ataxia consortium^28^ (http://enigma.ini.usc.edu/ongoing/enigma-ataxia/), based on the Spinal Cord Toolbox (SCT).^29^

All images were inspected to ensure coverage of at least the C2 vertebral level (or below), and to exclude for any additional pathology, in particular conditions causing spinal cord compression such as disc disease, myelopathy or tumour. To measure the CSA and eccentricity, we employed the SCT V.4.2.2.^29^ Briefly, we automatically segmented the cervical spinal cord using a deep-learning algorithm^30^ and visually inspected all segmentations for manual correction if necessary. Before registering the individual images to the PAM50 standard template,^31^ we manually marked the C2 and C3 vertebral levels at the posterior tip of the vertebral discs.^32 33^ The mean CSA and eccentricity were computed at each of the C1–C4 vertebral levels after correcting for the curvature of the spine. CSA is defined as the average number of pixels in the set of axial slices defining each vertebral level of the segmented spinal cord, and is reported in square millimetres (figure 1B). Eccentricity is computed by fitting an ellipse to each axial spinal slice and estimating the shortest and longest axis to determine the deviation of the ellipse from a perfect circle^28^ (higher values (closer to 1) indicate a more abnormal ellipsoid shape (ie, spinal flattening; figure 1)).

Since we used brain MRIs with limited spinal cord coverage, we were capable of assessing only the upper cervical spinal cord. Spinal cord coverage was slightly different across subjects due to field-of-view placement and head size variability, leading to different sample sizes for each vertebral level (SCA1—controls: C1=70, C2=70, C3=69 and C4=55; patients: C1=73, C2=73, C3=72 and C4=66; SCA2—controls: C1=85, C2=101, C3=98 and C4=67; patients: C1=93, C2=93, C3=91 and C4=70; SCA3—controls: C1=166, C2=178, C3=174 and C4=140; patients: C1=192, C2=192, C3=186 and C4=156; SCA6—controls: C1=49, C2=49, C3=48 and C4=40; patients: C1=54, C2=54, C3=53 and C4=40).

### Statistical analysis

All statistical analysis was done using the Matlab R2017b software (https://www.mathworks.com/products/matlab.html).

#### SCAs versus matched control group

We compared CSA and eccentricity at each vertebral level from C1 to C4 for all individuals with SCA1, SCA2, SCA3 and SCA6 relative to a SCA-specific age-matched and sex-matched control group using ANCOVA tests with age, sex and site as covariates of non-interest. All tests were corrected for multiple comparisons (Bonferroni-corrected p<0.05). We also computed effect sizes (ES) of all statistically significant results using the Cohen’s *d* formula. We considered effect size values of 0.2 as small, 0.5 as moderate, 0.8 as large and >1.2 as very large, according to established conventions.^34^

#### Correlation analysis

We used the Pearson correlation coefficient to assess associations between spinal cord morphometric data (CSA and eccentricity) and clinical parameters (disease duration and ataxia severity). The data were adjusted to account for age, sex and site effects using a linear model.

#### Disease progression

To examine spinal cord morphometry across different disease stages, we defined four subgroups according to the time since ataxia onset when each participant’s scan was acquired: <5 years, 5–10 years, 11–15 years and more than 15 years. These divisions are based on previous studies available in the literature^12 28^ and do not represent clinically determined cut-offs, but rather provide an intuitive means of quantitatively assessing and reporting changes in ES with disease progression. An additional subgroup defined as preataxic was also created and included subjects with SARA score <3 at the time of MRI acquisition.^23–25^ In each of these subgroups and for each of the four diseases, CSA and eccentricity were compared with a non-ataxic control group matched by age, sex and site. We also compared each SCA1, SCA2 and SCA3 subgroup with their respective preataxic subgroup to assess evidence for progressive degeneration. Group differences were assessed using ANCOVAs with age, sex and site as covariates, with Bonferroni corrections to account for multiple comparisons across vertebral levels.

In order to further explore the progressive patterns of degeneration of spinal cord morphometric data, we used the approach described by Faber and colleagues (2021).^13^ Briefly, we z-transformed both CSA and eccentricity based on the distribution of the data in the non-ataxic control cohort and plotted z values versus time from ataxia onset. Negative values for time since ataxia onset indicate the predicted time to future ataxia onset, calculated as described above.^23 27^ Linear and quadratic curves were fit to the data, and their relative fit was assessed by the R^2^ change.

## Results

Demographic, clinical and genetic data of all subjects with ataxia are shown in table 1. Due to the general consistency of results across the four vertebral levels, we report the results from vertebral level C2 in the main manuscript, in-line with previous MRI-based studies,^11–13 15 17 19^ and report the remaining vertebral levels in the supplemental material (online supplemental tables S7-S23).

### Individuals with SCA versus controls

**Figure 2:**
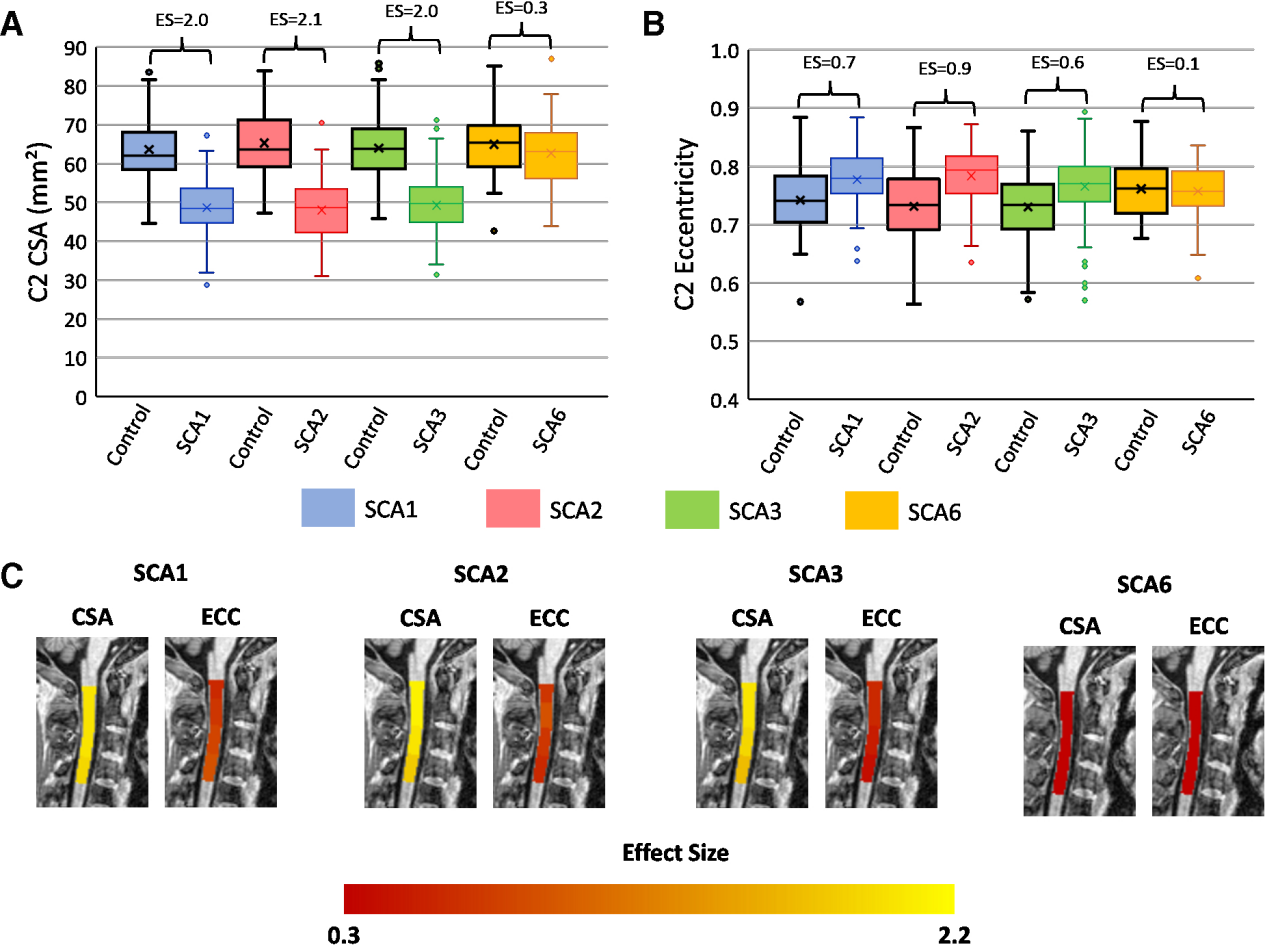
Box plots showing group differences at the C2 spinal cord level in each disease group relative to a matched control group (age-adjusted, sex-adjusted and site-adjusted). (A) C2 cross-sectional area in square millimetres; (B) C2 eccentricity; (C) visualisation of effect sizes overlaid on a spinal cord template image.

Individuals with SCA1, SCA2 and SCA3 had significantly reduced CSA at all vertebral levels with very large ES relative to controls (Cohen’s *d=*1.7–2.0; figure 2; online supplemental tables S7–S9). Similarly, we found significantly increased eccentricity at all vertebral levels (figure 2), although with substantially smaller ES in comparison to CSA (*d*=0.4–0.9; figure 2; online supplemental tables S7–S9). In contrast, in individuals with SCA6 relative to their respective control cohort, we did not observe significant CSA reduction (*d*=0.3) or increased eccentricity (*d*=0.1; figure 2; online supplemental table S10).

### Preataxic individuals with SCAs versus controls

**Figure 3:**
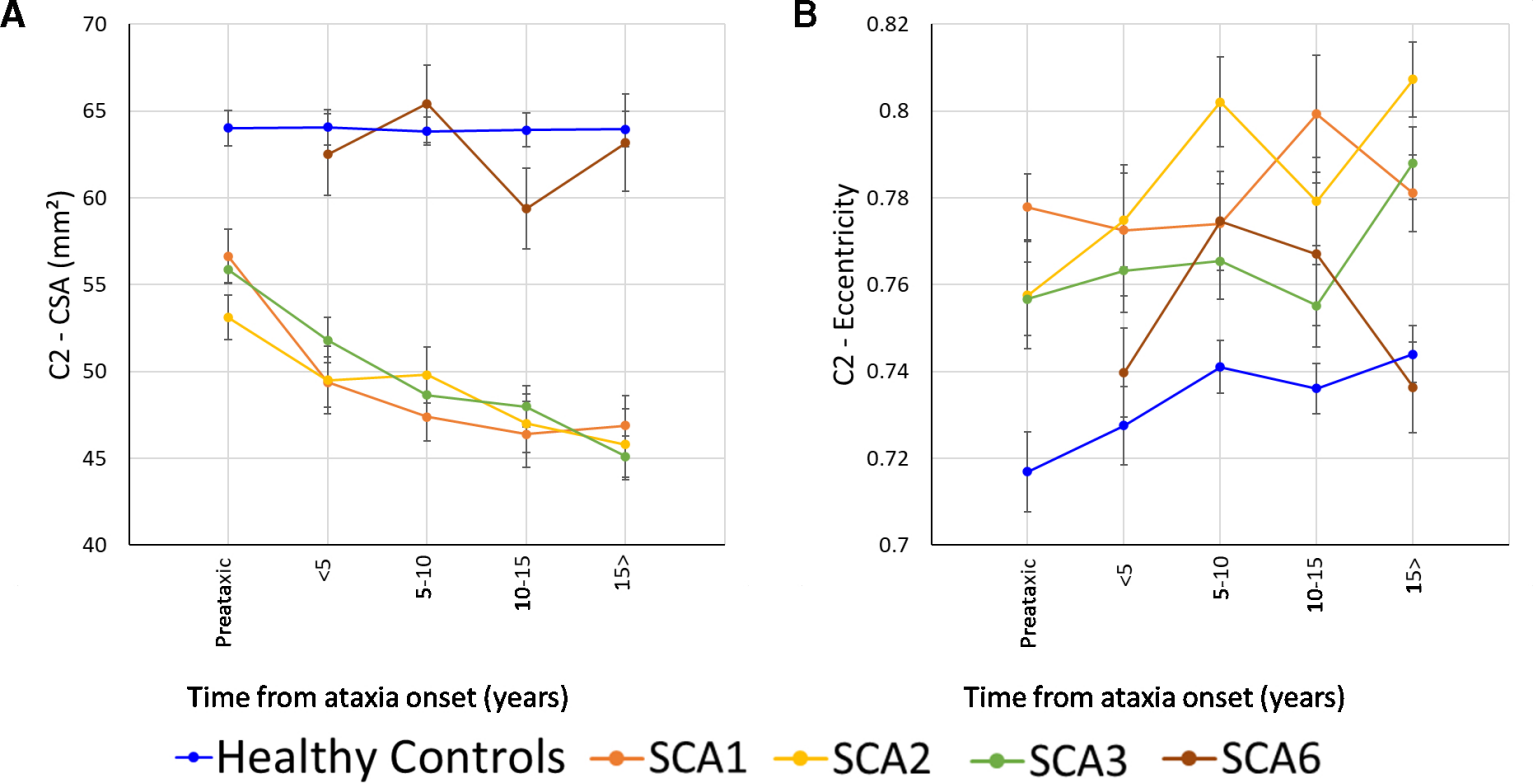
Results showing the progressive atrophy of the (A) C2 CSA and (B) C2 eccentricity in participants with SCA1 (orange), SCA2 (yellow), SCA3 (green) and SCA6 (brown). Subgroups are defined based on time since ataxia onset. Preataxic: subjects with Scale for Assessment and Rating of Ataxia score <3 at the time of MRI assessment.^29^ The healthy controls were age-matched, sex-matched and site-matched for each SCA subgroup, with the plotted datapoint representing the mean cervical spinal cord area or eccentricity of all controls included in each subgroup; error bars=SE error of the mean. CSA, cross-sectional area; SCA, spinocerebellar ataxia.

Mean time to ataxia onset ranged between −3.3 and −9.2 years in the three SCA groups. Preataxic individuals with SCA3 had significantly reduced CSA compared with controls at all vertebral levels with very large ES (figure 3, *d*=1.3–1.7; online supplemental table S13). The preataxic SCA2 cohort also had smaller CSA relative to their matched controls for vertebral levels C1–C3 (figure 3, *d*=1.4–1.8; online supplemental table S12). In contrast, we did not observe significant CSA reduction in preataxic individuals with SCA1 relative to their respective control cohort, despite having large ES (figure 3, *d*=0.8–1.2; online supplemental table S11), likely due to limited statistical power resulting from the small sample size (n=11).

Only the SCA2 cohort showed significantly higher eccentricity between preataxic individuals and controls, and only at the C2 level (*d*=1.1; online supplemental table S12). However, large ES were also evident for SCA1 (C2–C4, *d*=0.9–1.3; online supplemental table S15). Eccentricity was not different in the SCA3 preataxic group relative to controls (*d*=0.3–0.6; online supplemental table S13).

### Correlation analysis

**Figure 4:**
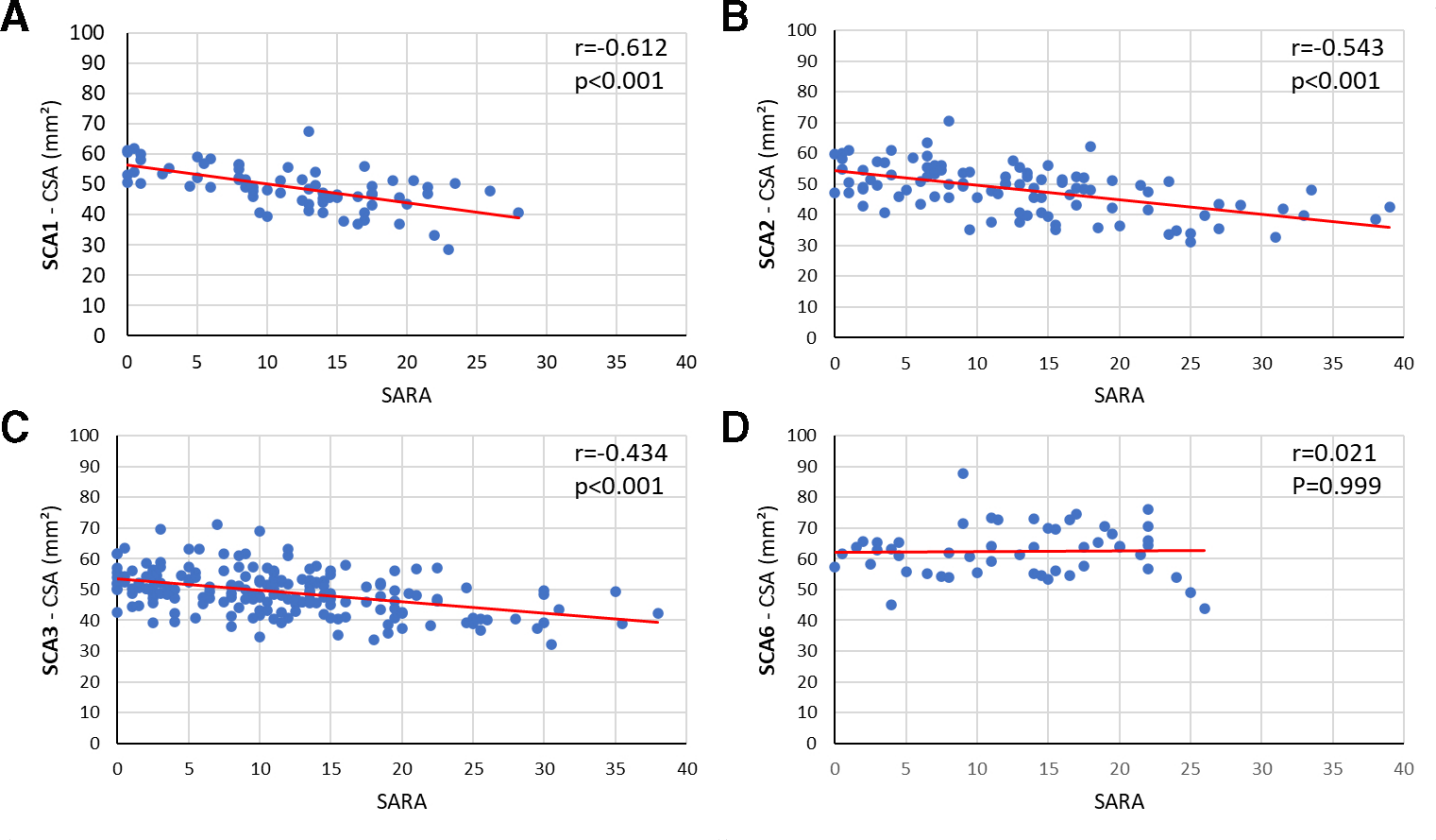
Correlations between CSA at the C2 level and SARA score for (A) SCA1, (B) SCA2, (C) SCA3 and (D) SCA6. CSA, cross-sectional area; SARA, Scale for Assessment and Rating of Ataxia; SCA, spinocerebellar ataxia.

As illustrated in figure 4, we found significant correlations between SARA (reflecting ataxia severity) and CSA at all vertebral levels for SCA1 (r=−0.61 to −0.62; online supplemental table S14), SCA2 (r=−0.41 to −0.61; online supplemental table S14) and SCA3 (r=−0.42 to −0.52; online supplemental table S14). Correlations in the SCA6 cohort were not significant (r=0.06 to −0.05; online supplemental table S14).

Correlations with time since ataxia onset were weaker, and only reached significance (Bonferroni corrected) for CSA at C1 in the SCA2 cohort (C1: r=−0.310, p=0.021) and CSA at all vertebral levels in the SCA3 cohort (C1: r=−0.219, p=0.015; C2: r=−0.209, p=0.022; C3: r=−0.313, p<0.001; C4: r=−0.238, p=0.016; online supplemental table S15).

In contrast, we did not find any significant correlations between eccentricity and SARA or ataxia duration, with exceptions of C1 and C3 in the SCA2 cohort (SARA—C1: r=0.277, p=0.024; C3: r=0.335, p=0.004 and time from ataxia onset—C1: r=0.288, p=0.038) and C4 in the SCA1 cohort (SARA—C4: r=0.424, p=0.002; online supplemental tables S14 and S15).

### Disease progression

The subgroup analyses based on time since ataxia onset showed that CSA was reduced relative to controls at all disease stages in SCA1, SCA2 and SCA3 (online supplemental tables S11–S13). CSA was also significantly smaller at all ataxic stages relative to preataxic patients for SCA1 and SCA3 groups at all vertebral levels (figure 3, online supplemental tables S16 and S18). Ataxic individuals with SCA2 showed significantly reduced CSA in the 10–15 years and 15+ years duration subgroups when compared with the preataxic cohort at the C1–C3 vertebral levels (figure 3, online supplemental table S17). There were no subgroup differences in SCA6 relative to controls or between disease stages (online supplemental tables S19 and S20).

Increased eccentricity, relative to controls, was only observed in late stages of the SCA1 and SCA3 cohorts (figure 3, online supplemental tables S11, S13, S16, S18). In contrast, SCA2 showed abnormal eccentricity in early stages of the disease with a progressive pattern of degeneration up to 10 years of disease duration (figure 3, online supplemental tables 12 and 17). There was no eccentricity staging effect for SCA6 (online supplemental tables S19 and S20).

**Figure 5:**
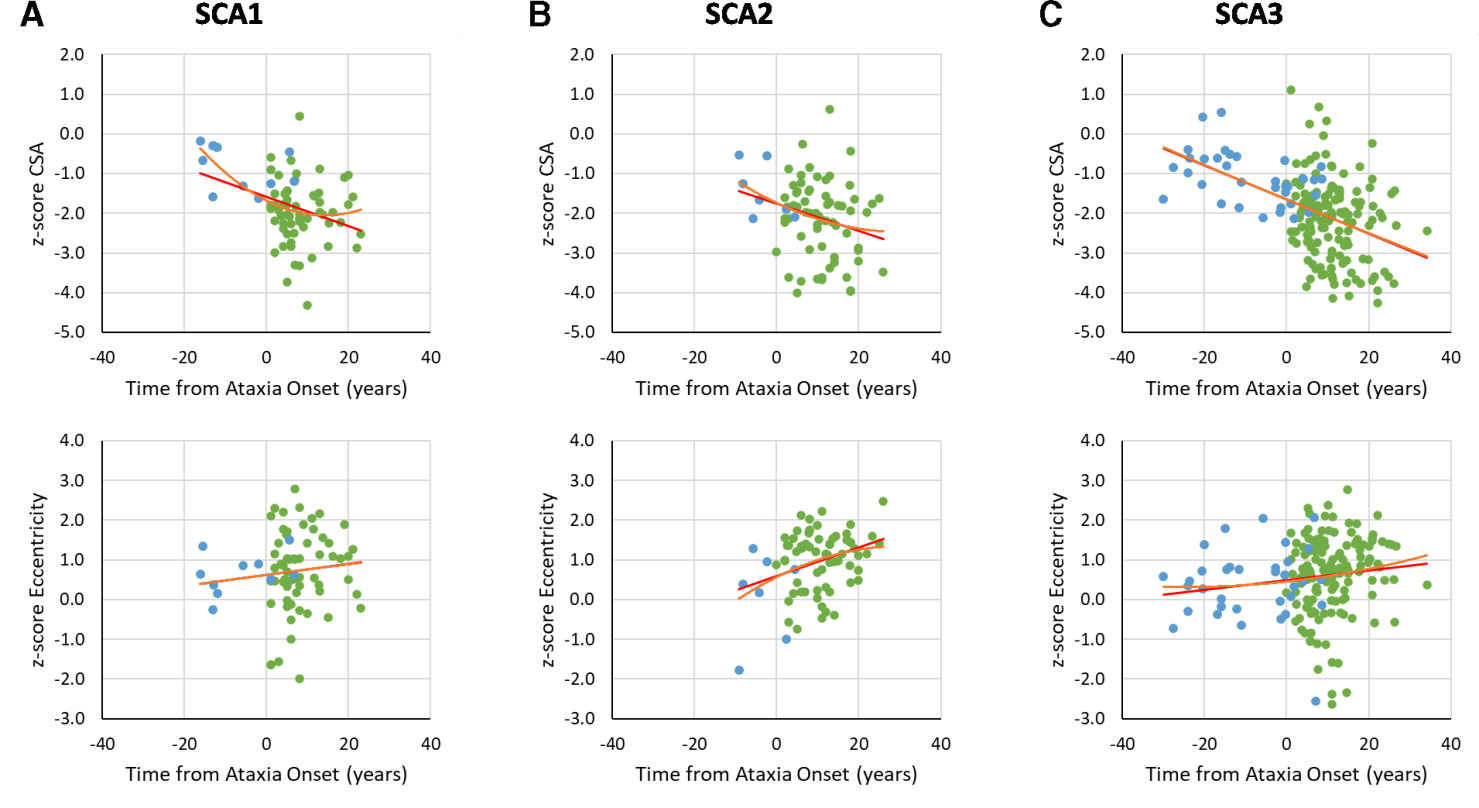
Graphs of z-transformed CSA or eccentricity at the C2 vertebral level versus time from ataxia onset (green). The negative values (blue) for disease duration indicate the predicted time to ataxia onset calculated using Tezenas formulas for SCA1 and SCA2^23^ and Peng formula for SCA3,^27^ based on CAG repeat length and current participant age. CSA, cross-sectional area; SCA, spinocerebellar ataxia.

Examination of the relationships between z-transformed CSA or eccentricity versus time since ataxia onset showed faster change of CSA over time in comparison to eccentricity. Indeed, the r^2^ of the CSA trend relative to the eccentricity trend was higher for patients with SCA1 and SCA3 (figure 5, online supplemental figures S1 and S2). SCA2 showed a different pattern, with eccentricity also showing evidence of significant evolution across the disease course (online supplemental tables S21–S23). In addition, we observed significantly improved model fit in SCA1 using a quadratic relative to a linear function for CSA at the C2 and C3 vertebrae (C2: R²-change=0.055, p=0.032; C3: R²-change=0.045, p=0.050), and eccentricity at the C4 level (R²-change=0.080, p=0.015) indicating a possible non-linear pattern of progression (online supplemental table S21). The SCA2 cohort also showed an improved fit using a quadratic function for eccentricity at the C3 and C4 vertebral levels (C3: R²-change=0.077, p=0.009; C4: R²-change=0.060, p=0.047), although this may have been driven by one outlier (figure 5; online supplemental table S22). Non-linear modelling did not improve the prediction of z-transformed spinal cord morphometric data versus time from ataxia onset in the SCA3 cohort (online supplemental table S23).

## Discussion

Spinal cord damage, although a well-defined pathological correlate of many SCAs, has not been robustly characterised in vivo in these diseases.^9–17 19^ In this study, we address this neglected aspect of SCAs by performing a comprehensive analysis of the upper spinal cord (C1–C4) anatomy using brain MRIs from the largest multisite cohort of individuals with genetic confirmation of SCA1, SCA2, SCA3 and SCA6 assembled so far. We found substantial CSA reduction with very large ES for all vertebral levels assessed in SCA1, SCA2 and SCA3, and significant correlations with ataxia severity and symptom duration. Eccentricity was also increased in these groups, although with substantially smaller ES relative to CSA, but correlated only with ataxia severity for the SCA2 cohort. Reduced CSA is already evident in preataxic individuals with SCA2 and SCA3—with similar trends in SCA1—and continues to decrease with disease progression. Eccentricity progresses only for individuals with SCA2. Spinal cord morphometry does not appear to be impacted in SCA6.

Our results for SCA1 and SCA3 are consistent with previous neuroimaging studies that found reduced CSA and anteroposterior flattening (ie, increased eccentricity) in patients relative to matched non-ataxic controls.^12 13 15^ One previous MRI study in SCA2 and SCA6 also reported spinal cord damage in SCA2, but preserved anteroposterior diameter of the spinal cord in individuals with SCA6.^19^ The pathological correlates of neuroimaging abnormalities reported herein are consistent with previous autopsy reports.^4 35–37^ Neuropathological studies have indeed described similar spinal cord micro-structural and macro-structural changes in SCA1, SCA2 and SCA3, including myelin loss and/or atrophy in the spinocerebellar tracts, dorsal columns and corticospinal tracts.^4 35–37^ In addition, spinal cord grey matter damage has also been reported in these three diseases, as shown by depletion of motor neurons in the ventral horns from cervical to lumbar regions.^4 35–37^ Taken together, these results indicate that the substrate for atrophy and flattening of the spinal cord likely involves both grey and white matter damage in SCA1, SCA2 and SCA3. Indeed, it would be interesting using an advanced spinal cord MRI protocol to assess both spinal cord grey and white matters in such SCA1, SCA2 and SCA3.^38^ In striking contrast, there were no morphometric spinal cord abnormalities in the SCA6 cohort. This is also in line with the available neuropathological studies that have reported damage essentially confined to Purkinje cells within the cerebellar cortex in these patients.^4 35–37^

Spinal cord damage is thus a hallmark of several SCAs, but the correlation between this damage and the clinical phenotype in this group of diseases remains elusive. We hypothesise that pyramidal signs and sensory deficits are the main clinical counterparts of spinal damage in SCA1, SCA2 and SCA3. This could not be formally tested in our analyses because detailed clinical information beyond SARA scores were not available for most sites. Despite that, supporting evidence comes from a recent study from the READISCA consortium, which reported sensory deficits to be conspicuous and precocious in both ataxic and preataxic SCA1 and SCA3 cohorts.^18^ Moreover, results from electrophysiological studies—particularly evoked potentials—are also supportive of our hypothesis. Indeed, patients with SCA1 typically present motor-evoked potentials with prolonged central conduction times, and patients with SCA1, SCA2 and SCA3 all present abnormal somatosensory-evoked potentials.^39^ In clinical practice, this indicates that symptoms of spinal cord damage (spasticity, lower limb weakness or sensory dysfunction) should be monitored from the earliest manifestations of the disease, and where practical, even prior to the onset of ataxia.

From a natural history point of view, our results indicate that spinal cord damage precedes the onset of clinical manifestations in SCA2 and SCA3. A previous single-site study found a very similar result for the SCA3 cohort.^12^ It is possible that this finding also holds for SCA1, but our cohort might have been underpowered (n=11) to confirm it statistically. The pseudo-longitudinal analyses carried out also suggest a progressive pattern of degeneration of the spinal cord in SCA1, SCA2 and SCA3. While the SCA1 group showed a severe CSA reduction in early stages of the disease, reaching a plateau after 5–10 years of disease duration, we observed a pattern of linear progression along the disease course for SCA2 and SCA3. Interestingly, only the SCA2 cohort showed a progressive increase in eccentricity, which is in line with the disease phenotype and course. This last parameter is considered a surrogate neuroimaging marker for dorsal column damage,^28^ corresponding in turn to deep sensory functions (proprioception and vibration sense). Even though sensory abnormalities due to dorsal root ganglia damage are well recognised in all three SCAs, they seem to appear earlier and to be more severe in SCA2.^39^ Indeed, Velázquez-Pérez *et al* (2014) report that sensory complaints and abnormal sensory nerve conduction studies are already present in a significant proportion of preataxic SCA2 carriers and progress over time.^40^

Our results not only contribute to the understanding of genotype–phenotype correlations in these SCAs, but also uncover potential MRI-based biomarkers for clinical use. In particular, CSA emerges as a potential tool for tracking progression in SCA1, SCA2 and SCA3, but not SCA6. This metric showed high correlation coefficients with disease severity and very high ES compared with non-ataxic individuals. In addition, CSA is already abnormal in premanifest stages of the diseases. The linear pattern of change over time in all subtypes suggest CSA might be a useful biomarker across all disease stages. Although compelling, we must be careful about these concepts, particularly because they arise from a cross-sectional investigation. However, this work makes it clear that prospective studies with large sample sizes (including preataxic individuals) and dedicated spinal cord MRI sequences must be undertaken to validate CSA as a neuroimaging biomarker for these SCAs.

To conclude, our data reveal that cervical spinal cord morphometric changes are present since preataxic stages of SCA1, SCA2 and SCA3 and progress with disease. In contrast, no spinal cord morphometric abnormality was found in SCA6. These results indicate that spinal cord MRI may be a useful marker of disease expression and progression in SCA1, SCA2 and SCA3.

## Data Availability

No data are available. All code and data processing instructions are available at https://github.com/Harding-Lab/enigma-ataxia.
