# Supplementary material for: Genotype-specific spinal cord damage in spinocerebellar ataxias: an ENIGMA-Ataxia study
Source: J Neurol Neurosurg Psychiatry. 2024 Feb 21;95(7):682–90. doi: 10.1136/jnnp-2023-332696 (PMC11187354; doi:10.1136/jnnp-2023-332696)
Supplement: Supplementary data [file jnnp-2023-332696supp001.pdf]

Supplementary Material

Supplementary Table 01: Demographics data of SCA1 patients and age, sex and site matched control group per site.

Controls						
	**Age (years)**	**Sex**		**CAG repeat length**	**SARA**	**Time from Ataxia Onset (years)**
		**Male**	**Female**			
**Campinas**	45.5±9.3	21	10	-	-	-
**Essen**	46.8±11.5	5	10	-	-	-
**Minnesota**	55.1±17.3	4	4	-	-	-
**Paris**	47.2±12.3	7	5	-	-	-
**Tubingen**	27.8±11.1	2	2	-	-	-
**Patients**						
	**Age (years)**	**Sex**		**CAG repeat length**	**SARA**	**Time from Ataxia Onset (years)**
		**Male**	**Female**			
**Campinas**	45.3±9.3	20	10	44.4±4.2	14.5±6.3	7.5±6.1
**Essen**	47.4±11.7	6	10	46.3±5.0	14.0±5.8	9.4±4.2
**Minnesota**	51.0±10.6	3	7	43.8±2.8	6.2±5.2	10.3±6.4
**Paris**	42.8±15.3	9	6	47.1±6.6	10.7±6.3	6.6±6.1
**Tubingen**	27.0±10.4	2	2	47.5±4.5	0.9±1.2	1.0±2.0

Supplementary Table 02: Demographics data of SCA2 patients and age, sex and site matched control group per site.

Controls						
	Age (years)	Sex		CAG repeat length	SARA	Time from Ataxia Onset (years)
		Male	Female			
**Campinas**	40.5±20.0	6	6	-	-	-
**Milan**	31.3±4.6	6	4	-	-	-
**Curitiba**	42.6±11.9	13	15	-	-	-
**Essen**	51.5±9.4	8	4	-	-	-
**Mexico**	41.6±14.0	7	9	-	-	-
**Minnesota**	55.1±17.3	4	4	-	-	-
**Paris**	42.3±11.4	5	5	-	-	-
**Tubingen**	33.8±8.5	2	2	-	-	-
**Patients**						
	Age (years)	Sex		CAG repeat length	SARA	Time from Ataxia Onset (years)
		Male	Female			
**Campinas**	41.1±18.8	8	6	45.9±10.5	18.7±13.2	8.8±5.7
**Milan**	46.6±9.1	1	9	41.2±3.1	13.4±6.4	11.8±4.7
**Curitiba**	45.2±11.6	8	9	41.7±2.9	14.5±6.1	11.5±5.2
**Essen**	52.1±9.8	10	4	39.8±2.1	11.7±10.8	10.8±7.8
**Mexico**	38.7±13.2	9	12	43.4±3.7	17.4±7.7	-
**Minnesota**	42.1±15.3	4	4	39.3±2.7	9.3±5.1	11.1±8.2
**Paris**	45.0±12.8	7	5	39.6±2.9	12.6±6.0	9.8±6.1
**Tubingen**	33.8±9.6	2	2	36.0±2.8	1.4±1.1	0.0±0.0

Supplementary Table 03: Demographics data of SCA3 patients and age, sex and site matched control group per site.

	Age (years)	Sex		CAG repeat length	SARA	Time from Ataxia Onset (years)
**Controls**						
		**Male**	**Female**			
**Campinas**	47.6±11.6	42	44	-	-	-
**Curitiba**	45.0±11.7	11	8	-	-	-
**Essen**	51.5±9.5	8	4	-	-	-
**Mexico**	43.7±12.8	5	7	-	-	-
**Minnesota**	55.1±17.3	4	4	-	-	-
**Nijmegen**	31.3±8.2	6	2	-	-	-
**Paris**	49.8±12.8	11	13	-	-	-
**Tubingen**	37.9±8.5	5	4	-	-	-
**Patients**						
	**Age (years)**	**Sex**		**CAG repeat length**	**SARA**	**Time from Ataxia Onset (years)**
		**Male**	**Female**			
**Campinas**	48.6±12.	39	46	71.8±3.5	13.3±8.6	10.2±6.4
**Curitiba**	46.1±12.5	11	8	-	14.1±5.6	11.9±7.5
**Essen**	53.0±13.7	13	11	68.4±4.1	12.0±8.1	14.5±7.4
**Mexico**	41.0±13.0	6	8	74.3±3.4	12.1±7.4	-
**Minnesota**	49.3±5.8	5	2	70.7±2.2	7.4±2.5	8.3±5.4
**Nijmegen**	37.4±9.6	9	8	66.8±2.9	1.5±1.0	0.0±0.0
**Paris**	51.6±12.3	7	10	68.3±5.8	12.2±7.5	8.0±4.6
**Tubingen**	40.4±8.3	6	3	67.6±2.4	3.3±4.0	2.0±4.4

Supplementary Table 04: Demographics data of SCA6 patients and age, sex and site matched control group per site.

**Controls**						
	**Age (years)**	**Sex**		**CAG repeat length**	**SARA**	**Time from Ataxia Onset (years)**
		**Male**	**Female**			
**Campinas**	64.8±5.3	9	5	-	-	-
**Essen**	60.4±12.5	15	9	-	-	-
**Minnesota**	55.1±17.3	4	4	-	-	-
**Tubingen**	56.3±9.1	3	0	-	-	-
**Patients**						
	**Age (years)**	**Sex**		**CAG repeat length**	**SARA**	**Time from Ataxia Onset (years)**
		**Male**	**Female**			
**Campinas**	67.9±7.9	10	7	-	13.9±6.3	14.1±7.0
**Essen**	61.6±10.9	15	10	22.4±1.3	13.3±7.0	11.3±6.3
**Minnesota**	65.8±8.3	2	6	22.4±1.5	13.1±8.1	13.4±6.7
**Tubingen**	55.7±7.5	3	0	21.7±0.6	1.3±1.3	2.7±4.6

Supplementary Table 05: Demographics data for all preataxic subjects.

		Average Age, y (range)	Sex, n (Male/Female)	CAG repeat length, long allele	Average SARA (range)	Average Time to Ataxia Onset, y (range)
**SCA1**	Controls (9)	35.8±13.0 (19-56)	3/6	-	-	-
	Patients (11)	35.4±12.3 (18-56)	3/8	44.9±3.7 (40-54)	0.8±0.9 (0-2.5)	-4.7±10.0 (-16.1-6.7)
**SCA2**	Controls (14)	34.2±9.4 (23-56)	8/6	-	-	-
	Patients (9)	32.7±8.9 (23-48)	4/5	37.8±3.0 (32-42)	1.2±0.9 (0-2.5)	-3.3±5.1 (-9.2-4.5)
**SCA3**	Controls (24)	38.2±9.8 (21-54)	12/12	-	-	-
	Patients (36)	39.0±9.1 (24-61)	15/21	70.3±4.3 (61-72)	11.3±8.2 (0-2.7)	-9.2±2.3 (-30.0-8.4)

Supplementary Table 06: Scanner and imaging acquisition details for each site.

	Site								
	**Campinas**	**Milan**	**Curitiba**	**Essen**	**México**	**Minnesota**	**Nijmegen**	**Paris**	**Tubingen**
**Scanner**	Philips Achieva	Philips Achieva	Siemens Skyra	Siemens Biograph	Philips Achieva	Siemens Trio and Prisma	Siemens Trio	Siemens Trio and Prisma	Siemens Trio
**Field strength (T)**	3	3	3	3	3	3	3	3	3
**Head coil (channels)**	8	32	16	16	32	32 (Trio)<br>64 (Prisma)	32	32 (Trio)<br>64 (Prisma)	32
**Sequence**	SPGR	SPGR	MPRAGE	MPRAGE	SPGR	MPRAGE	MPRAGE	MPRAGE	MPRAGE
**TR (ms)**	7	8	2530	2530	8	2530	2300	2530	2300
**TE (ms)**	3.201	3.5	3.36	3.26	3.7	3.65	3.03	3.65	3.51
**TI (ms)**	-	-	1100	1100	-	1100	1100	900	900
**Flip (deg)**	8	8	7	7		7	8	9	9
**Plane**	Sagittal	Sagittal	Sagittal	Sagittal	Sagittal	Coronal	Sagittal	Sagittal	Sagittal
**Slices**	180	160	176	176	160	224	192	160	176
**FOV (mm x mm)**	240x240	256x256	256 x 256	256x256	256x256	256x176	256x256	256x256	256x240
**Voxel Size (mm, x y z)**	1x1x1	1x1x1	1x1x1	1x1x1	1x1x1	1x1x1	1x1x1	1x1x1	1x1x1

Supplementary Table 07: Group differences between patients with SCA1 vs age, sex and site matched nonataxic control group.

CSA (mm^2)				
**Structures**	**Healthy Controls (mean±SD)**	**SCA1 (mean±SD)**	**p-value (Bonferroni)**	**Effect Size**
**C1**	65.6 ± 7.6	49.6 ± 8.1	<0.001	2.0
**C2**	63.6 ± 8.0	48.6 ± 7.2	<0.001	2.0
**C3**	62.7 ± 7.9	47.8 ± 7.1	<0.001	2.0
**C4**	63.7 ± 8.2	47.8 ± 8.3	<0.001	1.9
**Eccentricity**				
**Structures**	**Healthy Controls (mean±SD)**	**SCA1 (mean±SD)**	**p-value (Bonferroni)**	**Effect Size**
**C1**	0.693 ± 0.061	0.733 ± 0.069	0.002	0.6
**C2**	0.742 ± 0.051	0.777 ± 0.052	<0.001	0.7
**C3**	0.792 ± 0.044	0.824 ± 0.038	<0.001	0.8
**C4**	0.825 ± 0.037	0.857 ± 0.030	<0.001	0.9

Supplementary Table 08: Group differences between patients with SCA2 vs age, sex and site matched nonataxic control group.

CSA (mm^2)				
**Structures**	**Healthy Controls (mean±SD)**	**SCA2 (mean±SD)**	**p-value (Bonferroni)**	**Effect Size**
**C1**	67.4 ± 8.8	49.6 ± 8.2	<0.001	2.1
**C2**	65.3 ± 8.2	48.0 ± 7.9	<0.001	2.1
**C3**	63.9 ± 8.4	47.4 ± 8.3	<0.001	2.0
**C4**	63.8 ± 8.5	47.9 ± 8.8	<0.001	1.8
**Eccentricity**				
**Structures**	**Healthy Controls (mean±SD)**	**SCA2 (mean±SD)**	**p-value (Bonferroni)**	**Effect Size**
**C1**	0.692 ± 0.079	0.743 ± 0.070	<0.001	0.7
**C2**	0.731 ± 0.059	0.784 ± 0.049	<0.001	0.9
**C3**	0.781 ± 0.051	0.818 ± 0.050	<0.001	0.7
**C4**	0.825 ± 0.042	0.849 ± 0.042	0.004	0.6

Supplementary Table 09: Group differences between patients with SCA3 vs age, sex and site matched nonataxic control group.

CSA (mm²)				
**Structures**	**Healthy Controls (mean±SD)**	**SCA3 (mean±SD)**	**p-value (Bonferroni)**	**Effect Size**
**C1**	65.8 ± 7.9	50.3 ± 7.8	<0.001	2.0
**C2**	64.0 ± 7.5	49.3 ± 7.6	<0.001	2.0
**C3**	63.2 ± 7.6	48.6 ± 8.1	<0.001	1.9
**C4**	63.8 ± 7.9	49.9 ± 8.4	<0.001	1.7
**Eccentricity**				
**Structures**	**Healthy Controls (mean±SD)**	**SCA3 (mean±SD)**	**p-value (Bonferroni)**	**Effect Size**
**C1**	0.684 ± 0.073	0.728 ± 0.074	<0.001	0.6
**C2**	0.730 ± 0.058	0.765 ± 0.056	<0.001	0.6
**C3**	0.783 ± 0.050	0.809 ± 0.050	<0.001	0.5
**C4**	0.828 ± 0.040	0.844 ± 0.040	0.003	0.4

Supplementary Table 10: Group differences between patients with SCA6 vs age, sex and site matched nonataxic control group.

CSA (mm^2)				
**Structures**	**Healthy Controls (mean±SD)**	**SCA6 (mean±SD)**	**p-value (Bonferroni)**	**Effect Size**
**C1**	66.7 ± 10.3	63.0 ± 8.2	0.353	0.3
**C2**	65.1 ± 8.8	62.4 ± 8.8	0.757	0.3
**C3**	64.0 ± 9.1	60.7 ± 8.3	0.297	0.3
**C4**	65.1 ± 9.3	61.5 ± 8.9	0.451	0.3
**Eccentricity**				
**Structures**	**Healthy Controls (mean±SD)**	**SCA6 (mean±SD)**	**p-value (Bonferroni)**	**Effect Size**
**C1**	0.725 ± 0.058	0.728 ± 0.056	0.999	0.1
**C2**	0.761 ± 0.047	0.757 ± 0.046	0.999	0.1
**C3**	0.810 ± 0.039	0.798 ± 0.041	0.503	0.3
**C4**	0.837 ± 0.034	0.836 ± 0.029	0.999	0.1

Supplementary Table 11: Results of ROI-based analyses to assess spinal cord damage at each stage of SCA1. Subgroups based on disease duration, <5: Time from ataxia onset <5 years, 5-10: Time from ataxia onset between 5-10 years, 10-15: Time from ataxia onset between 10-15 years, 15>: Time from ataxia onset >15 years. Preataxic subjects present SARA score <3 during clinical and MRI assessment (Jacobi et al, 2013).

Vertebral Labels	Subgroup	Measures	Controls (mean±SD)	SCA1 (mean±SD)	p-value (Bonferroni)	Effect Size
C1	Preataxic	Area (mm²)	63.8 ± 8.1	57.9 ± 6.2	0.362	0.8
		ECC	0.696 ± 0.064	0.719 ± 0.070	0.999	0.3
	<5 years	Area (mm²)	65.5 ± 5.5	51.2 ± 6.5	<0.001	2.3
		ECC	0.673 ± 0.054	0.742 ± 0.068	0.036	1.1
	5-10 years	Area (mm²)	66.2 ± 7.8	48.3 ± 8.0	<0.001	2.3
		ECC	0.693 ± 0.061	0.729 ± 0.078	0.333	0.5
	10-15 years	Area (mm²)	63.7 ± 4.8	46.8 ± 7.2	<0.001	2.7
		ECC	0.689 ± 0.045	0.767 ± 0.047	0.010	1.7
	15> years	Area (mm²)	66.8 ± 8.2	46.7 ± 5.5	<0.001	2.9
		ECC	0.728 ± 0.051	0.732 ± 0.043	0.999	0.1
C2	Preataxic	Area (mm²)	62.5 ± 8.7	56.6 ± 5.2	0.346	0.8
		ECC	0.737 ± 0.059	0.778 ± 0.025	0.250	0.9
	<5 years	Area (mm²)	64.3 ± 7.6	49.1 ± 5.6	<0.001	2.3
		ECC	0.737 ± 0.042	0.775± 0.067	0.465	0.6
	5-10 years	Area (mm²)	63.6 ± 7.7	47.4 ± 7.2	<0.001	2.2
		ECC	0.748 ± 0.053	0.774 ± 0.053	0.340	0.5
	10-15 years	Area (mm²)	60.9 ± 5.7	46.4 ± 6.0	<0.001	2.5
		ECC	0.733 ± 0.050	0.799 ± 0.043	0.034	1.4
	15> years	Area (mm²)	65.4 ± 7.6	46.9 ± 5.4	<0.001	2.8
		ECC	0.756 ± 0.038	0.781 ± 0.028	0.442	0.8
C3	Preataxic	Area (mm²)	62.8 ± 8.8	56.1 ± 6.2	0.282	0.9
		ECC	0.764 ± 0.056	0.811 ± 0.023	0.103	1.1

	<5 years	Area (mm²)	63.5 ± 6.4	47.7 ± 5.6	<0.001	2.6
		ECC	0.787 ± 0.038	0.823 ± 0.048	0.143	0.9
	5-10 years	Area (mm²)	62.7 ± 8.4	46.2 ± 6.8	<0.001	2.2
		ECC	0.803 ± 0.042	0.825 ± 0.033	0.204	0.6
	10-15 years	Area (mm²)	59.8 ± 6.4	46.5 ± 6.0	0.001	2.2
		ECC	0.786 ± 0.039	0.839 ± 0.024	0.011	1.7
	15> years	Area (mm²)	62.8 ± 7.0	46.2 ± 6.1	<0.001	2.5
		ECC	0.800 ± 0.026	0.838 ± 0.034	0.067	1.2
**C4**	Preataxic	Area (mm²)	66.1 ± 8.7	56.9 ± 6.4	0.204	1.2
		ECC	0.798 ± 0.022	0.832 ± 0.032	0.181	1.3
	<5 years	Area (mm²)	63.9 ± 6.7	48.0 ± 6.8	<0.001	2.3
		ECC	0.814 ± 0.032	0.861 ± 0.030	0.001	1.5
	5-10 years	Area (mm²)	66.2 ± 8.1	46.8 ± 7.7	<0.001	2.5
		ECC	0.829 ± 0.040	0.860 ± 0.028	0.038	0.9
	10-15 years	Area (mm²)	59.7 ± 5.9	45.2 ± 9.5	0.014	1.8
		ECC	0.837 ± 0.034	0.866 ± 0.027	0.314	1.0
	15> years	Area (mm²)	58.3 ± 7.3	46.4 ± 9.1	0.034	1.4
		ECC	0.842 ± 0.031	0.859 ± 0.020	0.787	0.6

Supplementary Table 12: Results of ROI-based analyses to assess spinal cord damage at each stage of SCA2. Subgroups based on disease duration, <5: Time from ataxia onset <5 years, 5-10: Time from ataxia onset between 5-10 years, 10-15: Time from ataxia onset between 10-15 years, 15>: Time from ataxia onset >15 years. Preataxic subjects present SARA score <3 during clinical and MRI assessment (Jacobi et al, 2013).

Vertebral Labels	Subgroup	Measures	Controls (mean±SD)	SCA2 (mean±SD)	p-value (Bonferroni)	Effect Size
C1	Preataxic	Area (mm²)	65.7 ± 5.9	56.7 ± 5.9	0.003	1.6
		ECC	0.647 ± 0.090	0.708 ± 0.080	0.350	0.7
	<5 years	Area (mm²)	65.7 ± 6.8	51.4 ± 7.3	0.001	2.0
		ECC	0.651 ± 0.066	0.725 ± 0.054	0.046	1.2
	5-10 years	Area (mm²)	68.7 ± 9.3	51.4 ± 8.6	<0.001	2.4
		ECC	0.712 ± 0.085	0.763 ± 0.054	0.464	0.8
	10-15 years	Area (mm²)	68.5 ± 10.3	48.0 ± 7.8	<0.001	2.4
		ECC	0.702 ± 0.068	0.729 ± 0.082	0.999	0.4
	15> years	Area (mm²)	63.3 ± 8.4	46.7 ± 7.6	<0.001	2.2
		ECC	0.711 ± 0.076	0.780 ± 0.048	0.013	1.2
C2	Preataxic	Area (mm²)	63.5 ± 6.9	53.1 ± 5.9	0.001	1.6
		ECC	0.690 ± 0.058	0.757 ± 0.065	0.046	1.1
	<5 years	Area (mm²)	63.5 ± 7.6	49.5 ± 7.1	0.001	1.9
		ECC	0.704 ± 0.052	0.775 ± 0.039	0.009	1.6
	5-10 years	Area (mm²)	65.8 ± 8.0	50.0 ± 7.9	<0.001	2.3
		ECC	0.767 ± 0.044	0.802 ± 0.045	0.350	0.8
	10-15 years	Area (mm²)	67.2 ± 8.9	47.1 ± 8.6	<0.001	2.4
		ECC	0.741 ± 0.049	0.779 ± 0.047	0.062	0.8
	15> years	Area (mm²)	61.7 ± 8.8	45.7 ± 8.0	<0.001	2.0
		ECC	0.737 ± 0.078	0.807 ± 0.029	0.003	1.4
C3	Preataxic	Area (mm²)	62.6 ± 7.2	52.3 ± 8.2	0.001	1.4
		ECC	0.752 ± 0.063	0.773 ± 0.075	0.999	0.3

	<5 years	Area (mm²)	63.1 ± 8.0	50.1 ± 6.6	0.003	1.8
		ECC	0.753 ± 0.051	0.804 ± 0.026	0.036	1.3
	5-10 years	Area (mm²)	64.7 ± 8.4	49.0 ± 7.7	<0.001	2.2
		ECC	0.815 ± 0.026	0.838 ± 0.039	0.634	0.6
	10-15 years	Area (mm²)	65.1 ± 9.4	46.0 ± 8.4	<0.001	2.2
		ECC	0.793 ± 0.042	0.822 ± 0.037	0.129	0.8
	15> years	Area (mm²)	59.5 ± 8.3	45.3 ± 8.8	0.001	1.7
		ECC	0.798 ± 0.042	0.841 ± 0.023	0.006	1.3
**C4**	Preataxic	Area (mm²)	64.6 ± 8.6	49.4 ± 7.8	0.056	1.8
		ECC	0.814 ± 0.057	0.810 ± 0.055	0.999	0.1
	<5 years	Area (mm²)	62.6 ± 8.1	51.3 ± 6.4	0.008	1.6
		ECC	0.801 ± 0.053	0.847 ± 0.023	0.064	1.2
	5-10 years	Area (mm²)	65.4 ± 7.0	49.9 ± 8.9	<0.001	2.0
		ECC	0.842 ± 0.028	0.860 ± 0.035	0.999	0.5
	10-15 years	Area (mm²)	64.1 ± 9.0	45.7 ± 9.6	<0.001	2.1
		ECC	0.837 ± 0.029	0.861 ± 0.031	0.187	0.8
	15> years	Area (mm²)	59.5 ± 8.8	47.0 ± 10.2	0.053	1.3
		ECC	0.820 ± 0.034	0.862 ± 0.026	0.017	1.4

Supplementary Table 13: Results of ROI-based analyses to assess spinal cord damage at each stage of SCA3. Subgroups based on disease duration, <5: Time from ataxia onset <5 years, 5-10: Time from ataxia onset between 5-10 years, 10-15: Time from ataxia onset between 10-15 years, 15>: Time from ataxia onset >15 years. Preataxic subjects present SARA score <3 during clinical and MRI assessment (Jacobi et al, 2013).

**Vertebral Labels**	**Subgroup**	**Measures**	**Controls (mean±SD)**	**SCA3 (mean±SD)**	**p-value (Bonferroni)**	**Effect Size**
**C1**	Preataxic	Area (mm²)	65.6 ± 6.6	56.6 ± 5.5	<0.001	1.7
		ECC	0.673 ± 0.085	0.703 ± 0.079	0.742	0.4
	<5 years	Area (mm²)	65.3 ± 8.1	53.2 ± 6.7	<0.001	2.0
		ECC	0.689 ± 0.080	0.726± 0.049	0.433	0.6
	5-10 years	Area (mm²)	65.8 ± 7.5	49.8 ± 8.3	<0.001	2.1
		ECC	0.684 ± 0.065	0.727 ± 0.080	0.030	0.6
	10-15 years	Area (mm²)	65.5 ± 8.1	48.6 ± 6.6	<0.001	2.1
		ECC	0.683 ± 0.062	0.719 ± 0.071	0.089	0.5
	15> years	Area (mm²)	66.6± 9.2	45.9 ± 7.8	<0.001	2.4
		ECC	0.704± 0.067	0.766 ± 0.049	<0.001	1.1
**C2**	Preataxic	Area (mm²)	65.2 ± 7.0	55.6 ± 4.9	<0.001	1.7
		ECC	0.723 ± 0.066	0.757 ± 0.051	0.166	0.6
	<5 years	Area (mm²)	64.2 ± 7.8	51.9 ± 6.5	<0.001	2.0
		ECC	0.731 ± 0.074	0.764 ± 0.045	0.440	0.5
	5-10 years	Area (mm²)	63.4 ± 6.8	48.5 ± 7.8	<0.001	2.1
		ECC	0.729 ± 0.054	0.766 ± 0.059	0.019	0.6
	10-15 years	Area (mm²)	63.1 ± 7.7	47.7 ± 7.2	<0.001	2.0
		ECC	0.734 ± 0.048	0.755 ± 0.060	0.362	0.4
	15> years	Area (mm²)	64.6 ± 8.2	45.0 ± 7.5	<0.001	2.5
		ECC	0.743 ± 0.057	0.788 ± 0.051	0.002	0.9
**C3**	Preataxic	Area (mm²)	64.7 ± 7.4	55.7 ± 4.8	<0.001	1.6
		ECC	0.787 ± 0.045	0.803 ± 0.040	0.897	0.4

	<5 years	Area (mm^2)	62.3 ± 7.3	51.1 ± 7.1	<0.001	1.8
		ECC	0.779 ± 0.062	0.805 ± 0.041	0.557	0.5
	5-10 years	Area (mm^2)	62.7 ± 6.9	48.1 ± 7.9	<0.001	2.0
		ECC	0.780 ± 0.048	0.811 ± 0.047	0.025	0.6
	10-15 years	Area (mm^2)	61.9 ± 7.7	46.9 ± 7.4	<0.001	1.9
		ECC	0.790 ± 0.045	0.809 ± 0.051	0.398	0.4
	15> years	Area (mm^2)	64.0 ± 8.0	43.7 ± 8.3	<0.001	2.5
		ECC	0.793 ± 0.045	0.826 ± 0.047	0.019	0.7
**C4**	Preataxic	Area (mm^2)	65.2 ± 8.6	56.9 ± 5.3	0.001	1.3
		ECC	0.826 ± 0.035	0.836 ± 0.037	0.999	0.3
	<5 years	Area (mm^2)	62.5 ± 6.1	51.7 ± 7.6	<0.001	1.6
		ECC	0.827 ± 0.047	0.841 ± 0.031	0.999	0.4
	5-10 years	Area (mm^2)	63.7 ± 7.2	49.0 ± 8.8	<0.001	1.8
		ECC	0.823 ± 0.039	0.853 ± 0.045	0.025	0.7
	10-15 years	Area (mm^2)	63.2 ± 8.7	49.2 ± 7.7	<0.001	1.6
		ECC	0.833 ± 0.040	0.842 ± 0.036	0.999	0.2
	15> years	Area (mm^2)	64.1 ± 8.0	45.2 ± 8.4	<0.001	2.3
		ECC	0.840 ± 0.032	0.854 ± 0.038	0.568	0.4

Supplementary Table 14: Correlations between ataxia severity (SARA score) and CSA or eccentricity for individuals with SCA1, SCA2, SCA3 and SCA6.

CSA (mm²)								
	SCA1		SCA2		SCA3		SCA6	
	R	p-value	R	p-value	R	p-value	R	p-value
C1	-0.620	<0.001	-0.614	<0.001	-0.424	<0.001	0.064	0.999
C2	-0.612	<0.001	-0.543	<0.001	-0.434	<0.001	0.021	0.999
C3	-0.618	<0.001	-0.504	<0.001	-0.509	<0.001	-0.035	0.999
C4	-0.618	<0.001	-0.413	<0.001	-0.524	<0.001	-0.054	0.999
Eccentricity								
	SCA1		SCA2		SCA3		SCA6	
	R	p-value	R	p-value	R	p-value	R	p-value
C1	0.229	0.212	0.277	0.024	0.141	0.210	0.064	0.999
C2	0.173	0.581	0.228	0.099	0.118	0.426	0.021	0.999
C3	0.223	0.241	0.335	0.004	0.126	0.352	-0.035	0.999
C4	0.424	0.002	0.167	0.622	0.129	0.438	-0.054	0.999

Supplementary Table 15: Correlations between time from ataxia onset and CSA or eccentricity for individuals with SCA1, SCA2, SCA3 and SCA6.

CSA (mm²)								
	SCA1		SCA2		SCA3		SCA6	
	R	p-value	R	p-value	R	p-value	R	p-value
C1	-0.205	0.381	-0.310	0.021	-0.219	0.015	0.119	0.999
C2	-0.153	0.868	-0.238	0.133	-0.209	0.022	0.144	0.999
C3	-0.154	0.852	-0.180	0.462	-0.313	<0.001	0.093	0.999
C4	-0.173	0.712	-0.144	0.999	-0.238	0.016	0.166	0.999
Eccentricity								
	SCA1		SCA2		SCA3		SCA6	
	R	p-value	R	p-value	R	p-value	R	p-value
C1	0.171	0.668	0.288	0.038	0.118	0.479	0.119	0.999
C2	0.198	0.430	0.239	0.131	0.051	0.999	0.144	0.999
C3	0.152	0.874	0.267	0.072	0.017	0.999	0.093	0.999
C4	0.107	0.999	0.159	0.826	-0.015	0.999	0.166	0.999

Supplementary Table 16: Results of ROI-based analyses to assess progressive spinal cord damage at each stage of SCA1 relative to the preataxic cohort.

**CSA (mm²)**								
	**<5 years**		**5-10 years**		**10-15 years**		**15> years**	
	**p-value**	**ES**	**p-value**	**ES**	**p-value**	**ES**	**p-value**	**ES**
**C1**	0.023	1.0	0.001	1.3	0.001	1.3	<0.001	1.9
**C2**	0.008	1.3	<0.001	1.4	0.019	0.9	0.001	1.8
**C3**	0.010	1.4	0.001	1.5	0.032	0.8	0.002	1.6
**C4**	0.013	1.3	0.005	1.4	0.245	0.5	0.019	1.3
**Eccentricity**								
	**<5 years**		**5-10 years**		**10-15 years**		**15> years**	
	**p-value**	**ES**	**p-value**	**ES**	**p-value**	**ES**	**p-value**	**ES**
**C1**	0.598	0.3	0.661	0.1	0.449	0.3	0.621	0.2
**C2**	0.598	0.1	0.742	0.1	0.265	0.4	0.791	0.1
**C3**	0.268	0.3	0.134	0.4	0.011	1.0	0.058	0.9
**C4**	0.045	0.9	0.059	1.0	0.006	1.3	0.053	1.0

Supplementary Table 17: Results of ROI-based analyses to assess progressive spinal cord damage at each stage of SCA2 relative to the preataxic cohort.

CSA (mm^2)								
	<5 years		5-10 years		10-15 years		15> years	
	p-value	ES	p-value	ES	p-value	ES	p-value	ES
**C1**	0.058	0.8	0.029	0.8	0.001	1.3	0.001	1.3
**C2**	0.177	0.6	0.171	0.5	0.019	0.9	0.012	1.0
**C3**	0.402	0.4	0.166	0.5	0.032	0.8	0.024	0.9
**C4**	0.638	0.2	0.968	0.0	0.245	0.5	0.489	0.3
**Eccentricity**								
	<5 years		5-10 years		10-15 years		15> years	
	p-value	ES	p-value	ES	p-value	ES	p-value	ES
**C1**	0.557	0.2	0.029	0.8	0.449	0.3	0.005	1.1
**C2**	0.448	0.3	0.033	0.8	0.265	0.4	0.011	1.0
**C3**	0.176	0.6	0.003	1.2	0.011	1.0	0.001	1.4
**C4**	0.043	1.0	0.007	1.3	0.006	1.3	0.004	1.4

Supplementary Table 18: Results of ROI-based analyses to assess progressive spinal cord damage at each stage of SCA3 relative to the preataxic cohort.

CSA (mm²)								
	**<5 years**		**5-10 years**		**10-15 years**		**15> years**	
	**p-value**	**ES**	**p-value**	**ES**	**p-value**	**ES**	**p-value**	**ES**
**C1**	0.010	0.7	<0.001	1.1	<0.001	1.3	<0.001	1.7
**C2**	0.006	0.8	<0.001	1.1	<0.001	1.3	<0.001	1.7
**C3**	0.002	0.9	<0.001	1.2	<0.001	1.4	<0.001	1.8
**C4**	0.008	0.8	<0.001	1.0	<0.001	1.1	<0.001	1.6
**Eccentricity**								
	**<5 years**		**5-10 years**		**10-15 years**		**15> years**	
	**p-value**	**ES**	**p-value**	**ES**	**p-value**	**ES**	**p-value**	**ES**
**C1**	0.243	0.3	0.180	0.3	0.362	0.2	<0.001	1.0
**C2**	0.630	0.1	0.481	0.2	0.906	<0.1	0.011	0.6
**C3**	0.801	0.1	0.408	0.2	0.557	0.1	0.021	0.5
**C4**	0.647	0.1	0.108	0.4	0.564	0.2	0.076	0.5

Supplementary Table 19: Results of ROI-based analyses to assess spinal cord damage at each stage of SCA6. Subgroups based on disease duration, <5: Time from ataxia onset <5 years, 5-10: Time from ataxia onset between 5-10 years, 10-15: Time from ataxia onset between 10-15 years, 15>: Time from ataxia onset >15 years.

Vertebral Labels	Subgroup	Measures	Controls (mean±SD)	SCA3 (mean±SD)	p-value (Bonferroni)	Effect Size
**C1**	Preataxic	Area (mm²)	-	-	-	-
		ECC	-	-	-	-
	<5 years	Area (mm²)	66.6 ± 5.0	62.5 ± 7.5	0.859	0.6
		ECC	0.744 ± 0.068	0.705 ± 0.043	0.711	0.7
	5-10 years	Area (mm²)	62.7 ± 8.1	65.1 ± 8.8	0.667	0.3
		ECC	0.731 ± 0.064	0.758 ± 0.035	0.747	0.5
	10-15 years	Area (mm²)	64.7 ± 7.9	61.2 ± 8.7	0.999	0.4
		ECC	0.726 ± 0.037	0.724 ± 0.076	0.999	<0.1
	15> years	Area (mm²)	69.3 ± 10.5	64.5 ± 8.3	0.743	0.5
		ECC	0.712 ± 0.060	0.713 ± 0.047	0.999	<0.1
**C2**	Preataxic	Area (mm²)	-	-	-	-
		ECC	-	-	-	-
	<5 years	Area (mm²)	63.5 ± 5.0	62.6 ± 5.5	0.999	0.2
		ECC	0.768 ± 0.044	0.739 ± 0.027	0.481	0.8
	5-10 years	Area (mm²)	60.9 ± 7.4	65.3 ± 7.8	0.667	0.6
		ECC	0.773 ± 0.046	0.774 ± 0.042	0.999	<0.1
	10-15 years	Area (mm²)	63.8 ± 6.7	59.8 ± 7.3	0.768	0.6
		ECC	0.750 ± 0.038	0.768 ± 0.056	0.999	0.4
	15> years	Area (mm²)	69.1 ± 6.4	64.3 ± 9.7	0.565	0.6
		ECC	0.758 ± 0.043	0.737 ± 0.033	0.645	0.5
**C3**	Preataxic	Area (mm²)	-	-	-	-
		ECC	-	-	-	-
	<5 years	Area (mm²)	62.1 ± 6.4	62.1 ± 8.7	0.999	<0.1

		ECC	0.809 ± 0.033	0.778 ± 0.034	0.353	0.9
	5-10 years	Area (mm²)	61.7 ± 6.9	62.8 ± 8.1	0.999	0.2
		ECC	0.814 ± 0.034	0.816 ± 0.025	0.999	0.1
	10-15 years	Area (mm²)	62.3 ± 8.4	58.9 ± 6.2	0.999	0.5
		ECC	0.816 ± 0.037	0.800 ± 0.059	0.999	0.3
	15> years	Area (mm²)	66.7 ± 7.5	62.4 ± 9.0	0.760	0.5
		ECC	0.809 ± 0.035	0.789 ± 0.031	0.514	0.6
**C4**	Preataxic	Area (mm²)	-	-	-	-
		ECC	-	-	-	-
	<5 years	Area (mm²)	67.1 ± 6.6	57.6 ± 4.2	0.134	1.8
		ECC	0.837 ± 0.053	0.845 ± 0.024	0.999	0.2
	5-10 years	Area (mm²)	60.0 ± 9.6	62.9 ± 9.8	0.999	0.3
		ECC	0.839 ± 0.026	0.844 ± 0.031	0.999	0.2
	10-15 years	Area (mm²)	63.4 ± 9.2	61.7 ± 9.2	0.999	0.2
		ECC	0.847 ± 0.024	0.832 ± 0.031	0.938	0.6
	15> years	Area (mm²)	67.3 ± 8.7	62.7 ± 8.7	0.853	0.5
		ECC	0.839 ± 0.027	0.826 ± 0.028	0.999	0.5

Supplementary Table 20: Results of ROI-based analyses to assess progressive spinal cord damage at each stage of SCA6 relative to the cohort with time from ataxia onset <5 years.

CSA (mm²)								
	<5 years		5-10 years		10-15 years		15> years	
	p-value	ES	p-value	ES	p-value	ES	p-value	ES
**C1**	-	-	0.538	0.3	0.966	0.2	0.425	0.1
**C2**	-	-	0.389	0.4	0.808	0.4	0.331	0.1
**C3**	-	-	0.722	0.1	0.692	0.5	0.419	0.1
**C4**	-	-	0.166	0.5	0.303	0.4	0.206	0.5
**Eccentricity**								
	<5 years		5-10 years		10-15 years		15> years	
	p-value	ES	p-value	ES	p-value	ES	p-value	ES
**C1**	-	-	0.010	1.4	0.416	0.3	0.231	0.2
**C2**	-	-	0.053	0.9	0.216	0.6	0.531	0.1
**C3**	-	-	0.008	1.3	0.443	0.4	0.414	0.3
**C4**	-	-	0.875	0.1	0.591	0.5	0.446	0.8

Supplementary Table 21: Comparison of coefficients between linear and quadratic models for the graph of z-transformed CSA/eccentricity versus time from ataxia onset of the SCA1 cohort.

CSA (mm²)								
**Vertebral Level**	**Model**	**RMSE**	**R-Squared**	**Adjusted R-Squared**	**F-statistic**	**Model p-value**	**R-squared Change**	**p-value**
**C1**	Linear	0.960	0.129	0.116	10.2	0.002	0.030	0.112
	Quadratic	0.950	0.159	0.134	6.4	0.003		
**C2**	Linear	0.811	0.124	0.112	9.8	0.003	0.055	0.032
	Quadratic	0.791	0.179	0.155	7.4	0.001		
**C3**	Linear	0.845	0.134	0.122	10.7	0.002	0.045	0.050
	Quadratic	0.828	0.180	0.155	7.4	0.001		
**C4**	Linear	0.994	0.094	0.080	6.5	0.013	0.021	0.220
	Quadratic	0.991	0.115	0.086	4.0	0.023		
**Eccentricity**								
**Vertebral Level**	**Model**	**RMSE**	**R-Squared**	**Adjusted R-Squared**	**F-statistic**	**Model p-value**	**R-squared Change**	**p-value**
**C1**	Linear	1.147	0.004	-0.010	0.3	0.601	0.002	0.698
	Quadratic	1.154	0.006	-0.023	0.2	0.812		
**C2**	Linear	0.982	0.013	-0.002	0.9	0.348	<0.001	0.972
	Quadratic	0.989	0.013	-0.016	0.4	0.646		
**C3**	Linear	0.791	0.049	0.035	3.5	0.065	0.009	0.403
	Quadratic	0.793	0.058	0.030	2.1	0.131		
**C4**	Linear	0.765	0.074	0.059	5.0	0.028	0.080	0.015
	Quadratic	0.737	0.154	0.127	5.6	0.006		

Supplementary Table 22: Comparison coefficients between linear and quadratic models for the graph of z-transformed CSA/eccentricity versus time from ataxia onset of the SCA2 cohort.

CSA (mm²)								
**Vertebral Level**	**Model**	**RMSE**	**R-Squared**	**Adjusted R-Squared**	**F-statistic**	**Model p-value**	**R-squared Change**	**p-value**
**C1**	Linear	0.878	0.102	0.089	7.9	0.006	0.007	0.448
	Quadratic	0.881	0.109	0.083	4.2	0.019		
**C2**	Linear	0.952	0.067	0.054	5.0	0.028	0.004	0.583
	Quadratic	0.957	0.071	0.044	2.6	0.079		
**C3**	Linear	0.927	0.066	0.052	4.8	0.032	0.016	0.272
	Quadratic	0.926	0.082	0.054	3.0	0.058		
**C4**	Linear	1.059	0.025	0.007	1.4	0.240	0.011	0.427
	Quadratic	1.063	0.035	0.000	1.0	0.374		
**Eccentricity**								
**Vertebral Level**	**Model**	**RMSE**	**R-Squared**	**Adjusted R-Squared**	**F-statistic**	**Model p-value**	**R-squared Change**	**p-value**
**C1**	Linear	0.812	0.077	0.064	5.8	0.018	0.003	0.619
	Quadratic	0.817	0.080	0.053	3.0	0.056		
**C2**	Linear	0.769	0.110	0.097	8.7	0.004	0.006	0.471
	Quadratic	0.772	0.117	0.091	4.6	0.014		
**C3**	Linear	0.824	0.178	0.166	14.7	<0.001	0.077	0.009
	Quadratic	0.790	0.255	0.232	11.4	<0.001		
**C4**	Linear	0.767	0.083	0.066	5.0	0.029	0.060	0.047
	Quadratic	0.748	0.143	0.112	4.6	0.014		

Supplementary Table 23: Comparison coefficients between linear and quadratic models for the graph of z-transformed CSA/eccentricity versus time from ataxia onset of the SCA3 cohort.

**CSA (mm²)**								
**Vertebral Level**	**Model**	**RMSE**	**R-Squared**	**Adjusted R-Squared**	**F-statistic**	**Model p-value**	**R-squared Change**	**p-value**
**C1**	Linear	0.899	0.212	0.208	46.6	<0.001	0.002	0.507
	Quadratic	0.901	0.214	0.205	23.5	<0.001		
**C2**	Linear	0.940	0.201	0.197	43.6	<0.001	<0.001	0.934
	Quadratic	0.943	0.201	0.192	21.7	<0.001		
**C3**	Linear	0.975	0.235	0.231	51.3	<0.001	<0.001	0.862
	Quadratic	0.977	0.235	0.226	25.5	<0.001		
**C4**	Linear	1.124	0.147	0.141	25.0	<0.001	<0.001	0.895
	Quadratic	1.127	0.147	0.135	12.4	<0.001		
**Eccentricity**								
**Vertebral Level**	**Model**	**RMSE**	**R-Squared**	**Adjusted R-Squared**	**F-statistic**	**Model p-value**	**R-squared Change**	**p-value**
**C1**	Linear	0.990	0.079	0.074	14.9	<0.001	<0.001	0.981
	Quadratic	0.993	0.079	0.069	7.4	0.001		
**C2**	Linear	0.958	0.019	0.013	3.3	0.072	0.002	0.523
	Quadratic	0.960	0.021	0.010	1.8	0.162		
**C3**	Linear	0.956	0.034	0.029	5.9	0.016	<0.001	0.973
	Quadratic	0.959	0.034	0.023	3.0	0.055		
**C4**	Linear	1.000	0.014	0.007	2.0	0.160	0.001	0.663
	Quadratic	1.003	0.015	0.001	1.1	0.341		

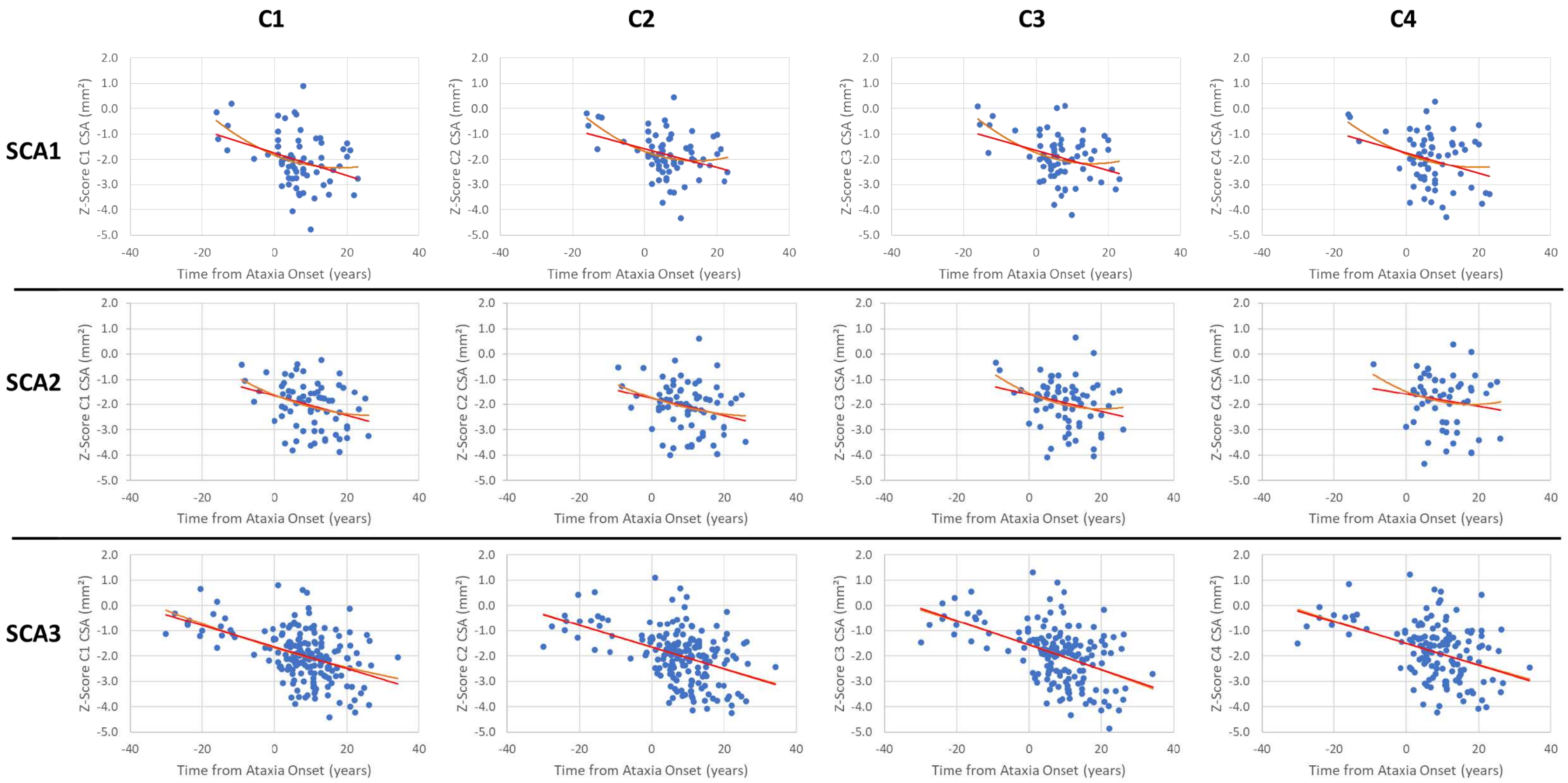

Supplementary Figure 01: Graphs of z-transformed CSA versus time from ataxia onset. The negative values for disease duration indicate the predicted time to ataxia onset calculated using Tezenas formulas for SCA1 and SCA2 (Tezenas du Montcel et al, 2014) and Peng formula for SCA3 (Peng et al, 2021), both are based on CAG repeat length and current participant age.

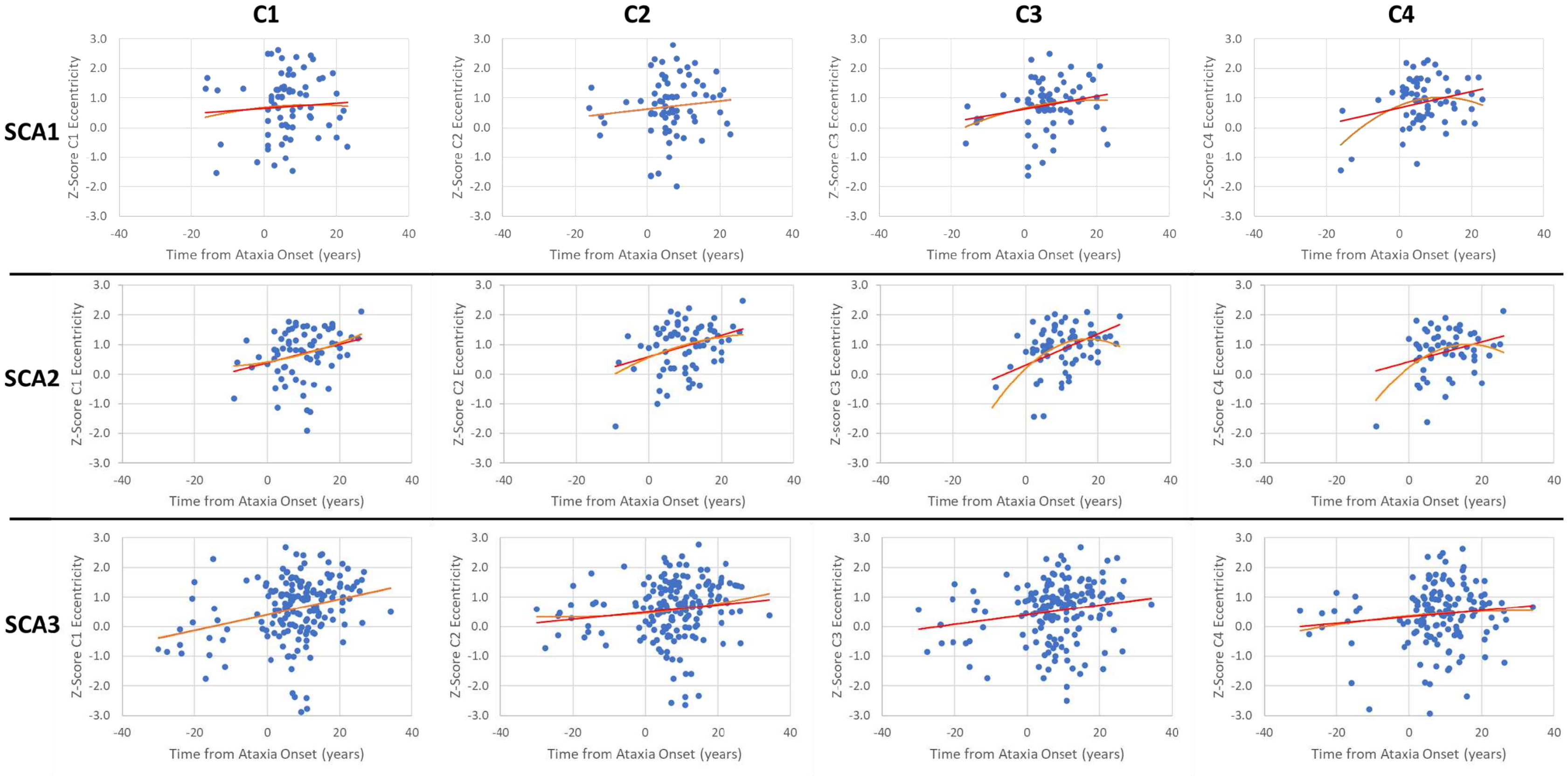

Supplementary Figure 02: Graphs of z-transformed Eccentricity versus time from ataxia onset. The negative values for disease duration indicate the predicted time to ataxia onset calculated using Tezenas formulas for SCA1 and SCA2 (Tezenas du Montcel et al, 2014) and Peng formula for SCA3 (Peng et al, 2021), both are based on CAG repeat length and current participant age.
